# Epidemiologic Investigation of Intestinal Parasite Infection and Associated Risk Factors among Primary Schoolchildren in the Manzini and Lubombo Provinces, the Kingdom of Eswatini

**DOI:** 10.1155/2022/9190333

**Published:** 2022-11-14

**Authors:** Ai-Wen Yin, Yueh-Lun Lee, Sindisiwe Dlamini, Gugu Maphalala, Chien-Wei Liao, Chia-Kwung Fan

**Affiliations:** ^1^Graduate Institute of Medical Sciences, College of Medicine, Taipei Medical University, 110 Taipei, Taiwan; ^2^Department of Molecular Parasitology and Tropical Diseases, School of Medicine, College of Medicine, Taipei Medical University, 110 Taipei, Taiwan; ^3^Department of Microbiology and Immunology, School of Medicine, College of Medicine, Taipei Medical University, 110 Taipei, Taiwan; ^4^National Health Laboratory Service, Ministry of Health, Mbabane, Eswatini; ^5^National Blood Transfusion Services, Ministry of Health, Mbabane, Eswatini; ^6^Research Center of International Tropical Medicine, College of Medicine, Taipei Medical University, 110 Taipei, Taiwan

## Abstract

Although the deworming program has been executed since 2000, the intestinal parasitic infection (IPI) rates among primary schoolchildren (PSC) in the two provinces of the Kingdom of Eswatini investigated in 2010 remained high, reaching 32.2%. In this study, we monitored the IPI status along with the associated risk factors for PSC in two provinces—Manzini and Lubombo. After consent from their parents/guardians, a total of 316 samples collected from PSC with grades 1 to 3 from four primary schools in Manzini and Lubombo were examined by the Merthiolate-Iodine-Formaldehyde (MIF) method. In addition, demographic characteristics and risk factors acquired by questionnaire surveys were included to be statistically analyzed. The overall prevalence was 40.5% (128/316), of which the infection rate in Manzini and Lubombo was 28.8% (19/66) and 58.3% (74/140), respectively. Pathogenic protozoa had the highest infection rate of 20.6% (65/316), including *Entamoeba histolytica/dispar* (8.5%, 27/316), *Giardia duodenalis* (14.6%, 46/316), and *Blastocystis hominis* (9.8%, 31/316). In terms of helminth infection, the infection rate was quite low, 1.6% only, and these five infected cases included four cases of *Hymenolepis nana* and one case of *Enterobius vermicularis* infection. Present study showed that 27.8% (88/316) of PSC were infected by more than one pathogenic parasite. Personal hygiene like washing hands before a meal has a significant protection effect (OR = 0.32, 95% CI = 0.14–0.75, *p*=0.009). Rain or well water and the type of water supply from which they drank also showed a considerable risk factor (OR = 2.44, 95% CI = 1.25–4.79, *p*=0.04). The IPI rate in PSC seems unlikely changed compared to that of the previous survey conducted in 2010, especially when the pathogenic protozoan infection rate remains high. Treatment of infected PSC with appropriate medication to reduce intestinal pathogenic protozoan infection should be seriously considered by Eswatini Health Authority.

## 1. Introduction

Intestinal parasitic infections (IPIs) influence one-third of the world population [1], and millions of people suffer from both helminths and protozoa due to poor personal hygiene and lack of clean water or proper sewage disposal [2]. Several studies showed that a more severe situation and worm burden occur in school-age children [3, 4]. Persistent infections may lead to chronic malnutrition and iron deficiency anemia that can cause lower study performance [5]. IPIs consisted of both helminths and protozoan infections, including mainly *Ascaris lumbricoides,*over 819 million people were infected and followed by *Trichuris trichiura* (464.6 million) and then hookworms (*Ancylostoma duodenale* and *Necator americanus*) (438.9 million) [6], also referred to as soil-transmitted helminths (STHs), while pathogenic protozoan infections include *Entamoeba histolytica* (48 million), *Giardia intestinalis* (2.8 million), and *Blastocystis hominis* [7].

Although the deworming program has been executed since 2000, the outcome of this campaign remained largely unclear due to no monitoring of its effectiveness. In 2010, we analyzed a survey focusing on IPIs among primary schoolchildren (PSC) and we found that the overall IPI rate was 32.2% (86/267), of which the infection rate of helminths was quite low (0.4%, 1/267) [8]. Only one primary schoolchild was infected by one helminthic parasite—*A. lumbricoides*. Nevertheless, the pathogenic protozoan infection rate, in contrast, was pretty high, reaching 20.6% (55/267). Among the pathogenic protozoan infections, the main species were *Ent. histolytica/dispar*, *B. hominis*, and *G. intestinalis*, whereas the main species of non-pathogenic protozoa were *Ent. coli*, *Endolimax nana*, *Iodoentamoeba butschlii*, and *Chilomastix mesnili* [8].

The main purpose of this study was to monitor the IPI prevalence and treatment efficiency in PSC after an annual regular deworming campaign for 10 years. In addition, IPI status in spatial distribution in two different altitudes was also investigated.

## 2. Methods

### 2.1. The Geography Description of the Kingdom of Eswatini

**Figure 1:**
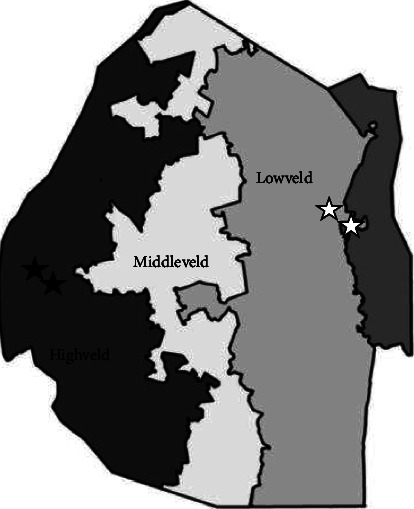
Primary schools' localities in Highveld and Lowveld regions of Manzini and Lubombo provinces, the Kingdom of Eswatini.

Eswatini is surrounded by South Africa in the north, west, and south and Mozambique in the east. Determined by altitude, Eswatini is divided into four regions with diverse landscapes. From west to east, the average altitude descended and separated Eswatini into three different areas. The capital, Mbabane, is located on Highveld with an average altitude of 1,200 meters. An average 700 meters high area is called Middleveld and the largest city is Manzini and Lowveld, mainly about 250 meters is less populated than the other areas (Figure 1).

### 2.2. Study Population and Subject Selection

The sample size was estimated using the general formula, *n* = *z*^2^*p* (1 − *p*)/d^2^, under 95% confidence level, 5% margin of error, and 21% response distribution, and getting at least 285 samples is recommended [9]. According to the geographic locality, four elementary schools located in two different regions, Lowveld and Highveld, were selected by Bilharzia and Deworming Program, Eswatini (Figure 1). Seventy-five PSC from grades 1 to 3 from each school would be recruited, and ideally a total of 300 students should participate in the present study.

### 2.3. Diagnostics and Assessment of Risk Factors Associated with IPIs and Treatment

Present study was undertaken from July 19 to August 10, 2019. To compare (IPI) outcomes and associated risk factors, we first designed a questionnaire and items included personal basic data (student's gender, family members, and parents' occupation) and hygiene status including washing hands after using toilet facilities, washing hands before eating, fingernail trimming, finger sucking, the way and frequency of bathing, the type of floor, and the frequency of cleaning the bedding. Microscopic examination of eggs or cysts or trophozoites in feces was performed by using the Merthiolate-Iodine-Formaldehyde (MIF) method (Shin-Yung Co., Ltd., Taiwan) which is routinely conducted in Taiwan CDC and Hospitals. The excellence of MIF method is capable of reducing the interference by stool particles, dismissing the disgusting smells during fecal specimen processing and particularly important, to improve the identification efficiency of intestinal helminthic eggs and protozoan cysts or trophozoites in clinical laboratory practice [10]. Each primary schoolchild was given a wide-opening container for stool collection and the stool specimen was immediately transported to Parasitology Laboratory of Mbabane Government Hospital for further examination. Stool samples containing eggs, cysts, or trophozoites as detected by the MIF method were considered positive, and infected PSC were treated appropriately by physicians in the respective province hospital.

### 2.4. Statistical Analysis

Differences in the prevalence of infection based on independent variables including gender, age group, residence, and parents' employment status were determined by a chi-squared (*X*^2^) test. The univariate crude odds ratio (OR) and 95% confidence interval (CI) were used to determine associations between independent variables, risk factors, and infection, and *p* value of <0.05 was considered statistically significant. All statistical analysis of data from the questionnaire and parasitological examination were performed using SAS version 9.3 software (SAS Institute, Cary, NC, USA) [11].

### 2.5. Ethical Consideration

Informed consent was obtained from every parent/guardian who agreed with their kids to participate in the present study, and approval was granted by the Eswatini Health and Human Research Review Board (SHR172/2019).

## 3. Results

After consent from parents/guardians, although 380 PSC were recruited, only 316 samples from 201 boys and 115 girls, whose ages ranged from 5 to 15 years (mean age of 8.00 ± 1.62 years), were valid to be used for further analysis because they gave their fecal specimens along with complete questionnaire data. Overall, 128 IPI cases were detected among 316 subjects, thus resulting in the prevalence rate of 40.5%. Age group of 8–9 y old had the highest prevalence of 44.09% (56/127), followed by 41.04% (55/134) in the age group of ≦7 y old and then 33.33% (17/51) in the age group of ≧10 y old. The prevalence by gender showed that boys had 39.30% (79/201) and girls had 42.61% (49/115). The prevalence of PSC who lived in Lowveld (Lubombo) area was 37.28% (63/169) and those who lived in Highveld (Manzini) area had prevalence of 42.22% (65/147) (Table 1). The prevalence of PSC whose parents were both unemployed was 45.71% (16/35), followed by 43.33% (52/120) in both had jobs and 37.27% (60/161) in either parent hired.

The prevalence of PSC who lived in brick houses was 42.98% (98/228) and that of PSC who lived in mud houses was 36.62% (26/71) (Table 1).

Single infection was the most commonly found among PSC (28.48%, 90/316), followed by dual infection of 8.86% (28/316) and multiple infection was the least found (2.85% (9/316)). The prevalence of boys in Lowveld and Highveld was 33.60% (36/109) and 45.70% (43/94), respectively, while the figure in girls was 43.55% (26/62) and 41.50% (22/53), respectively (Table 2). Totally, nine different intestinal parasites including two helminths, e.g., *E. vermicularis* and *H. nana*, while three pathogenic protozoa including *Ent. histolytica/dispar, G. intestinalis*, *and B. hominis* and four non-pathogenic protozoa including *Ent. coli*, *E. nana*, *I. buetschii,* and *E. hartmanni* had been detected in the present study*. E. coli, G. duodenalis,* and *B. hominis* were the top three with high infection rate of 18.7% (59/316), 14.6% (46/316), and 9.8% (31/316), respectively (Table 2).

Crude logistic regression analysis showed that rain water and other types of water supply had a significant relevance in the infection rate (OR = 2.37, 95% CI = 1.19–4.66, *p*=0.04), compared to tap water. Personal hygiene like washing hands before meal showed a considerable protection effect (OR = 0.39, 95% CI = 0.16–0.95, *p*=0.04). In different residences, Highveld area showed more risk than Lowveld for PSC in the acquisition of pathogenic parasites (OR = 1.90, 95% CI = 1.05–3.41, *p*=0.03) (Table 3).

## 4. Discussion

Neglected tropical diseases (NTDs) have been formally recognized as targets for global action towards the Sustainable Development Goals (SDGs) aiming to end the epidemics of HIV, tuberculosis, malaria, and NTDs by 2030 [12]. The present study intended to help Eswatini Health Ministry to monitor the outcome of the annual deworming campaign regarding pathogenic IPIs among PSC through mutual collaboration with Public Health Department to fulfill the missions of SDG 3 and SDG 17. Among NTDs, IPIs including soil-transmitted helminthiasis (Ascariasis, Hookworm Diseases, Trichuriasis, and Strongyloidiasis) and protozoan infections (Amebiasis and Giardiasis) have influenced 3.5 billion persons and caused clinical morbidity in nearly 450 million people [7]. Furthermore, over 600 million school-aged children were vulnerable due to IPIs [7].

Present prevalence (40.5%) was lower than that of PSC in Ethiopia (52%) [13], Burkina Faso (60.8%) [14], Cote d'Ivoire (55.2%) [15], Nigeria (86.2%) [16], São Tomé and Príncipe (64.7%) [17], and Pakistan (52.8%) [18], while it was higher than that in Western Saudi Arabia (12%) [19], Iran (21.5%) [20], India (27.5%) [20], Nepal (31.5%) [21], Marshall Islands (22.8%) [22], and Senegal (35%) [23]. The difference in prevalence can be attributed to the following reasons: the timing of the survey due to deworming schedule, environmental conditions of the target areas, or contamination of water supplies [24].

Prevalence of IPIs in the present study was 40.5%, indicating no significant difference between the present and previous studies (41.9%) of 10 years ago. Helminth infection rate was still low, and this achievement may be due to the regular biannual deworming program whereby each primary schoolchild was given one tablet of albendazole or praziquantel for STHs or Bilharzia control [25]. Nevertheless, pathogenic protozoan infections seem likely long ignored, thus resulting in a still high infection rate.

This situation did not change and improve as such the infection status was similar to that found in a survey which was conducted by our team 10 years ago.

Substantial studies in different countries have also found similar situation that helminth infections have been greatly reduced, but pathogenic protozoan infections like *Ent. Histolytica/dispar*, *B. hominis,* or *G. intestinalis* are predominant IPIs [26]. It has been well known that amoebas, *B. hominis*, and *G. intestinalis* are able to cause gastrointestinal problems of varying severity and outcomes, including rendering children unable to attend school due to diarrhea or severe abdominal pain, consequently leading to low academic performance [27, 28]. It is time to address the importance as how to prevent PSC from pathogenic protozoan infections as well as treatment regimen [29].

Noteworthy, the predominant helminth infection in the present study was*H. nana* (4/5). Some studies have found that *H. nana* infection has a high prevalence in PSC and 50–75 million cases were estimated globally [30]. Although the symptom of *H. nana* infection is usually benign or asymptomatic [31–33], anemia and eosinophilia have been often reported [32]. Strikingly, a report indicated that *H. nana* in a HIV-infected man causes malignancy because transformed cells derived from *H. nana*may develop with morphologic features and invasive behavior compatible with cancer [34]. Although extra-intestinal*H. nana* infections are rare, cysticercoids have been reported in whole-blood preparations from glucocorticoid-treated children [35] and a case of fatal invasive *H. nana* infection with atypical morphologic features was described in a HIV-positive man [36, 37]. Moreover, *H. nana* is known to develop abnormal, enlarged, and ballooned cysticercoids in immunosuppressed mice [38]. Since HIV infection in Eswatini is very common [39], whether *H. nana* infection may cause malignancy in immunocompromised patients is a serious concern.

The association of age with infection was not significant, indicating that all age groups had almost equal exposure opportunity. This could be as a result of the general contamination of the environment, even though younger children are generally reported to have higher prevalence due to their poor personal hygiene like geophagia/pica [40]. Prevalence seemed likely to exist in different genders in different ecological environments in the present study, e.g., boys in Lowveld showed lower prevalence than girls, while the situation in Highveld was opposite. It could be explained that the environmental contamination by intestinal parasites is serious, thus making PSC susceptible to acquire the IPIs through frequent exposure to the contaminated environments, regardless of the different ecological environments. Moreover, on the whole, boys and girls had similar prevalence, so the infection was not gender-dependent in this study, which is consistent with other studies [16, 22], indicating boys or girls had equal opportunity in acquisition of IPIs. The occupation of parents/guardians also did not show a significant association with infection in the logistic analysis and such a similar finding was also recorded in Nigeria [16]. However, living in Highveld area than Lowveld area had significantly higher risk of acquisition of pathogenic parasites by logistic regression analysis. In Eswatini, annual rainfall was recorded highest in the Highveld, between 1,000 and 2,000 mm, while in the Lowveld, 500 to 900 mm per annum was recorded. The Highveld temperature is temperate and seldom uncomfortably hot, while the Lowveld may record temperatures around 40°C in summer [41]. Alternatively, it can be explained that more abundant rain water with temperate temperature in Highveld than Lowveld may provide more suitable environments for parasites to survive plus a lack of good sanitation. Also, contact with contaminated soil and infected water when taking care of body hygiene and domestic activities may lead to higher opportunities for PSC to acquire these parasites via fecal-oral route.

Therefore, regardless of living in different ecological environments, key measures would be to educate PSC the better personal hygienic habits as well as improve environmental sanitation to prevent intestinal pathogenic protozoan and helminthic infections. Also, proper treatment of infected PSC by considering adding praziquantel and/or metronidazole to mebendazole- or albendazole along with regular deworming regimens to Eswatini PSC is urgently needed.

## Figures and Tables

**Table 1:** Demographic characteristics and intestinal parasitic infections among primary schoolchildren from Manzini and Lubombo provinces, the Kingdom of Eswatini.

	Total examined (*N* = 316)	Number and prevalence (%)		
		Helminth (*N* = 5)	Protozoa (*N* = 126)	All (*N* = 128)
Age (years)				
≦7	134	4 (2.99)	53 (39.55)	55 (41.04)
8–9	127	1 (0.64)	56 (44.09)	56 (44.09)
≧10	51	0 (0)	17 (33.33)	17 (33.33)
Unknown	4	0	0	0
Gender				
Boys	201	2 (1.0)	79 (39.3)	79 (39.3)
Girls	115	3 (2.61)	47 (40.87)	49 (42.61)
Residence				
Lowveld	169	3 (1.78)	63 (37.28)	63 (37.28)
Highveld	147	2 (1.36)	63 (42.86)	65 (42.22)
Employment of parents				
Both	120	2 (1.67)	52 (43.33)	52 (43.33)
Either	161	3 (1.86)	58 (36.02)	60 (37.27)
None	35	0 (0)	16 (45.71)	16 (45.71)
Living environment				
Brick house	230	3 (1.32)	97 (42.17)	98 (42.61)
Mud house	71	2 (2.82)	25 (35.21)	26 (36.62)
Hut	4	0	2 (50.00)	2 (50.00)
Wooden house	7	0	0	0
Homeless	1	0	1 (100)	1 (100)
Unknown	3	0	1 (33.33)	1 (33.33)

**Table 2:** Polyparasitism status among primary schoolchildren from Manzini and Lubombo provinces, the Kingdom of Eswatini.

Area	Status of intestinal parasite infection			Subtotal (%)
	Single (%)	Dual (%)	Multiple (%)	
Lowveld				
Boys (*N* = 107)	24 (22.4)	12 (11.2)	0 (0)	36 (33.6)
Girls (*N* = 62)	22 (35.5)	4 (6.5)	1 (1.6)	27 (43.5)
Highveld				
Boys (*N* = 94)	31 (33.0)	8 (8.5)	4 (4.3)	43 (45.7)
Girls (*N* = 53)	13 (24.5)	5 (9.4)	4 (7.5)	22 (41.5)
Residence				
Lowveld (*N* = 169)	46 (27.2)	16 (9.5)	1 (0.6)	63 (37.3)
Highveld (*N* = 147)	44 (29.9)	13 (8.8)	8 (5.4)	65 (44.2)
Helminth				
*E. vermicularis*	0 (0)	1 (0.3)	0 (0)	1 (0.3)
*H. nana*	2 (0.6)	2 (0.6)	0 (0)	4 (1.3)
Pathogenic protozoa				
*E. histolytica/dispar*	10 (3.2)	12 (3.8)	5 (1.6)	27 (8.5)
*G. duodenalis*	29 (9.2)	11 (3.5)	6 (1.9)	46 (14.6)
*B. hominis*	15 (4.7)	10 (3.2)	6 (1.9)	31 (9.8)
Non-pathogenic protozoa				
*E. coli*	32 (10.1)	18 (5.7)	9 (2.8)	59 (18.7)
*E. nana*	0 (0)	1 (0.3)	1 (0.3)	2 (0.6)
*I. buetschlii*	2 (0.6)	1 (0.3)	2 (0.6)	5 (1.6)
*E. hartmanni*	0 (0)	0 (0)	3 (0.9)	3 (0.9)
Total	90 (28.5)	56 (17.7)	32 (10.1)	178 (56.3)

**Table 3:** Logistic regression analysis of intestinal pathogenic parasitic infections among primary schoolchildren from Manzini and Lubombo provinces, the Kingdom of Eswatini.

Variables	Prevalence					
	All			Pathogenic parasites		
	OR	95% CI	*p* value	OR	95% CI	*p* value
Age (y)						
≦7	1.00			1.00		
8-9	1.61	0.90–2.88	0.21	1.31	0.70–2.46	0.69
≧10	1.27	0.57–2.83	0.99	1.35	0.57–3.21	0.68
Gender						
Boys	1.00					
Girls	1.14	0.66–1.97	0.65	1.45	0.79–2.62	0.22
Residence						
Lowveld	1.00			1.00		
Highveld	1.49	0.66–1.97	0.16	**1.90**	**1.05–3.41**	**0.03**
Employment of parents						
None	1.00			1.00		
Either	0.58	0.23–1.45	0.08	0.62	0.23–1.66	0.19
Both	1.01	0.74–2.49	0.38	0.92	0.34–2.48	0.63
Living environment						
Others	1.00			1.00		
Brick house	1.36	0.74–1.61	0.31	1.17	0.61–2.26	0.63
Raw meat						
No	1.00			1.00		
Yes	0.78	0.38–1.61	0.51	1.14	0.53–2.43	0.74
Raw vegetable						
No	1.00			1.00		
Yes	0.39	0.12–1.28	0.12	0.66	0.18–2.42	0.53
Water supply						
Tap water	1.00			1.00		
Well	1.68	0.88–3.18	0.76	1.62	0.78–3.27	0.67
Rain and others	**2.37**	**1.19–4.66**	**0.04**	2.01	0.97–4.17	0.16
Eating ground						
No	1.00			1.00		
Yes	1.21	0.54–2.69	0.65	1.65	0.68–4.01	0.27
Washing hands before meal						
No	1.00			1.00		
Yes	0.52	0.22–1.22	0.13	0.39	**0.16–0.95**	**0.04**
Washing hands after toilet						
No	1.00			1.00		
Yes	1.44	0.61–3.41	0.41	1.62	0.63–4.18	0.31
Wearing shoes						
No	1.00			1.00		
Yes	1.61	0.91–2.82	0.10	1.58	0.86–2.94	0.14
Touching soil						
No	1.00			1.00		
Yes	0.99	0.55–1.81	0.99	0.86	0.45–1.64	0.58
Pet						
No	1.00			1.00		
Yes	1.47	0.69–3.15	0.32	1.26	0.55–2.91	0.58

## Data Availability

The raw data can be acquired upon request.

## References

[1] WHO (2007). *Partners for Parasite Control: Geographical Distribution and Useful Facts and Stats*.

[2] Kantzanou M., Karalexi M. A., Vrioni G., Tsakris A. (2021). Prevalence of intestinal parasitic infections among children in Europe over the last five years. *Tropical Medicine and Infectious Disease*.

[3] Tegen D., Damtie D. (2021). Prevalence and risk factors associated with intestinal parasitic infection among primary school children in Dera district, northwest Ethiopia. *The Canadian Journal of Infectious Diseases & Medical Microbiology*.

[4] Montresor A., Crompton D. W. T., Gyorkos T. W., World Health Organization, Savioli L. (2002). *Helminth Control in School-Age Children: A Guide for Managers of Control Programmes*.

[5] Ngui R., Lim Y. A. L., Chong Kin L., Sek Chuen C., Jaffar S. (2012). Association between anaemia, iron deficiency anaemia, neglected parasitic infections, and socioeconomic factors in rural children of West Malaysia. *PLoS Neglected Tropical Diseases*.

[6] Ojha S. C., Jaide C., Jinawath N., Rotjanapan P., Baral P. (2014). Geohelminths: public health significance. *The Journal of Infection in Developing Countries*.

[7] WHO (2010). *Working to Overcome the Global Impact of Neglected Tropical Diseases: First WHO Report on Neglected Tropical Diseases*.

[8] Fan C. K., Liao C. W., Lyu S. Y. (2012). Prevalence of intestinal parasitic infections among primary schoolchildren in areas devoid of sanitation in northwestern Kingdom of Swaziland, Southern Africa. *Pathogens and Global Health*.

[9] Naing L., Winn T., Rusli B. N. (2006). Practical issues in calculating the sample size for prevalence studies. *Archives of orofacial Sciences*.

[10] Hsieh M. H., Lin W. Y., Dai C. Y. (2010). Intestinal parasitic infection detected by stool examination in foreign laborers in Kaohsiung. *The Kaohsiung Journal of Medical Sciences*.

[11] Fan C. K., Chuang T. W., Huang Y. C. (2019). Enterobius vermicularis infection: prevalence and risk factors among preschool children in kindergarten in the capital area, Republic of the Marshall Islands. *BMC Infectious Diseases*.

[12] Molyneux D. H., Savioli L., Engels D. (2017). Neglected tropical diseases: progress towards addressing the chronic pandemic. *The Lancet*.

[13] Chelkeba L., Mekonnen Z., Alemu Y., Emana D. (2020). Epidemiology of intestinal parasitic infections in preschool and school-aged Ethiopian children: a systematic review and meta-analysis. *BMC Public Health*.

[14] Ouermi D., Karou D. S., Ouattara I. (2012). Prevalence of intestinal parasites at Saint-Camille medical center in Ouagadougou (Burkina Faso), 1991 to 2010. *Médecine et Santé Tropicales*.

[15] Adoubryn K. D., Kouadio-Yapo C. G., Ouhon J., Aka N., Bintto F., Assoumou A. (2012). Intestinal parasites in children in Biankouma, Ivory Coast (mountaineous western region): efficacy and safety of praziquantel and albendazole. *Médecine et Santé Tropicales*.

[16] Gyang V. P., Chuang T. W., Liao C. W. (2019). Intestinal parasitic infections: current status and associated risk factors among school aged children in an archetypal African urban slum in Nigeria. *Journal of Microbiology, Immunology, and Infection*.

[17] Liao C. W., Fu C. J., Kao C. Y. (2016). Prevalence of intestinal parasitic infections among school children in capital areas of the Democratic Republic of São Tomé and Príncipe, West Africa. *African Health Sciences*.

[18] Mehraj V., Hatcher J., Akhtar S., Rafique G., Beg M. A. (2008). Prevalence and factors associated with intestinal parasitic infection among children in an urban slum of Karachi. *PLoS One*.

[19] Ka I. (2018). Prevalence of intestinal parasitic infection among school children in Taif. *Insights Biomed*.

[20] Bahrami F., Haghighi A., Zamini G., Khadem-Erfan M. B., Azargashb E. (2018). Prevalence and associated risk factors of intestinal parasitic infections in Kurdistan province, northwest Iran. *Cogent Medicine*.

[21] Sah R. B., Yadav S., Baral R., Bhattarai S., Jha N., Pokharel P. (2013). A study of prevalence of intestinal parasites and associated risk factors among the school children of Itahari, Eastern Region of Nepal. *Tropical parasitology*.

[22] Liao C. W., Chuang T. W., Huang Y. C. (2017). Intestinal parasitic infections: current prevalence and risk factors among schoolchildren in capital area of the Republic of Marshall Islands. *Acta Tropica*.

[23] Sylla K., Tine R. C. K., Sow D. (2018). Epidemiological profile of intestinal parasitic infection among preschool and school children living in a rural community in Senegal: a cross-sectional survey. *Journal of Bacteriology & Parasitology*.

[24] Elmonir W., Elaadli H., Amer A. (2021). Prevalence of intestinal parasitic infections and their associated risk factors among preschool and school children in Egypt. *PLoS One*.

[25] Makadzange K. (2018). *Eswatini Deworms at Least 85% of School-Aged Children*.

[26] Hajissa K., Islam M. A., Sanyang A. M., Mohamed Z. (2022). Prevalence of intestinal protozoan parasites among school children in africa: a systematic review and meta-analysis. *PLoS Neglected Tropical Diseases*.

[27] Müller I., Yap P., Steinmann P. (2016). Intestinal parasites, growth and physical fitness of schoolchildren in poor neighbourhoods of Port Elizabeth, South Africa: a cross-sectional survey. *Parasites & Vectors*.

[28] Sayasone S., Utzinger J., Akkhavong K., Odermatt P. (2015). Multiparasitism and intensity of helminth infections in relation to symptoms and nutritional status among children: a cross-sectional study in southern Lao People’s Democratic Republic. *Acta Tropica*.

[29] Turkeltaub J. A., McCarty T. R., Hotez P. J. (2015). The intestinal protozoa: emerging impact on global health and development. *Current Opinion in Gastroenterology*.

[30] Craig P., Ito A. (2007). Intestinal cestodes. *Current Opinion in Infectious Diseases*.

[31] Cabada M. M., Morales M. L., Lopez M. (2016). *Hymenolepis nana* impact among children in the highlands of Cusco, Peru: an emerging neglected parasite infection. *The American Journal of Tropical Medicine and Hygiene*.

[32] Willcocks B., McAuliffe G. N., Baird R. W. (2015). Dwarf tapeworm (*H ymenolepis nana*): characteristics in the northern territory 2002-2013. *Journal of Paediatrics and Child Health*.

[33] Abdel Hamid M. M., Eljack I. A., Osman M. K. M., Elaagip A. H., Muneer M. S. (2015). The prevalence of *Hymenolepis nana* among preschool children of displacement communities in Khartoum state, Sudan: a cross-sectional study. *Travel Medicine and Infectious Disease*.

[34] Muehlenbachs A., Bhatnagar J., Agudelo C. A. (2015). Malignant transformation of *Hymenolepis nana* in a human host. *New England Journal of Medicine*.

[35] Gamal-Eddin F. M., Aboul-Atta A. M., Hassounah O. A. (1986). Extra-intestinal nana cysticercoidiasis in asthmatic and filarised Egyptian patients. *Journal of the Egyptian Society of Parasitology*.

[36] Santamaría-Fríes M., Fajardo L. F., Sogin M. L., Olson P., Relman D. (1996). Lethal infection by a previously unrecognised metazoan parasite. *The Lancet*.

[37] Olson P. D., Yoder K., Fajardo L. F. L.-G. (2003). Lethal invasive cestodiasis in immunosuppressed patients. *The Journal of Infectious Diseases*.

[38] Lucas S. B., Hassounah O., Muller R., Doenhoff M. J. (1980). Abnormal development of hymenolepis nana larvae in immunosuppressed mice. *Journal of Helminthology*.

[39] Justman J., Reed J. B., Bicego G. (2017). Swaziland HIV incidence measurement survey (SHIMS): a prospective national cohort study. *The Lancet HIV*.

[40] Goodman D., Haji H. J., Bickle Q. D. (2007). A comparison of methods for detecting the eggs of ascaris, trichuris, and hookworm in infant stool, and the epidemiology of infection in zanzibari infants. *The American Journal of Tropical Medicine and Hygiene*.

[41] Mganu Manyatsi A., Zwane N., Dlamini M. (2015). Evaluation of satellite rainfall estimates for Swaziland. *American Journal of Agriculture and Forestry*.

